# Venus: Elucidating the Impact of Amino Acid Variants on Protein Function Beyond Structure Destabilisation

**DOI:** 10.1016/j.jmb.2022.167567

**Published:** 2022-06-15

**Authors:** Matteo P. Ferla, Alistair T. Pagnamenta, Leonidas Koukouflis, Jenny C. Taylor, Brian D. Marsden

**Affiliations:** 1 Wellcome Centre for Human Genetics, University of Oxford, Oxford OX3 7BN, UK; 2 Oxford NIHR Biomedical Research Centre, Oxford, UK; 3 Centre for Medicines Discovery, University of Oxford, Old Road Campus Research Building, Oxford OX3 7DQ, UK; 4 Kennedy Institute of Rheumatology, University of Oxford, Oxford OX3 7FY, UK

## Abstract

Exploring the functional effect of a non-synonymous coding variant at the protein level requires multiple pieces of information to be interpreted appropriately. This is particularly important when embarking on the study of a potentially pathogenic variant linked to a rare or monogenic disease. Whereas accurate protein stability predictions alone are generally informative, other effects, such as disruption of post-translational modifications or weakened ligand binding, may also contribute to the disease phenotype. Furthermore, consideration of nearby variants that are found in the healthy population may strengthen or refute a given mechanistic hypothesis. Whilst there are several bioinformatics tools available that score a genetic variant in terms of deleteriousness, there is no single tool that assembles multiple effects of a variant on the encoded protein, beyond structural stability, and presents them on the structure for inspection.

Venus is a web application which, given a protein substitution, rapidly estimates the predicted effect on protein stability of the variant, flags if the variant affects a post-translational modification site, a predicted linear motif or known annotation, and determines the effect on protein stability of variants which affect nearby residues and have been identified in healthy populations. Venus is built upon Michelanglo and the results can be exported to it, allowing them to be annotated and shared with other researchers.

Venus is freely accessible at https://venus.cmd.ox.ac.uk and its source code is openly available at https://github.com/CMD-Oxford/Michelanglo-and-Venus.

## Highlights

• Understanding how a genomic variant relates to pathogenicity is critical.
• Protein destabilisation, alone, is often not a plausible explanation.
• Nearby gnomAD variants and Uniprot annotations are often crucial for the hypothesis.
• We have developed the Venus webapp to help formulate potential hypotheses.
• Venus incorporates different pieces of information mapped onto structure.

## Graphical abstract

**Figure:**
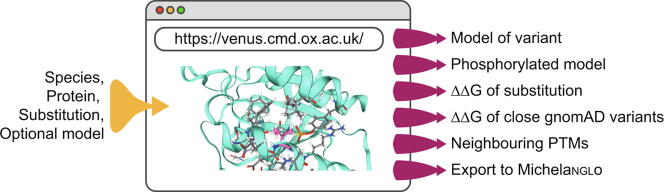

## Introduction

### Background

Whole genome sequencing (WGS) is increasingly being used in a clinical setting to provide genetic diagnoses for patients with rare disease.^1^, ^2^, ^3^, ^4^ However, assessing the mechanism of pathogenicity of variants identified from WGS is still not straightforward. Although empirical evidence of a variant’s effect on protein function is ultimately required to confirm pathogenicity, detailed annotation of variants at the genetic and protein level can greatly assist in the prioritization of variants for such functional studies. Since the majority of the pathogenic variants identified to date are coding variants, the impact of a specific variant on structure or predicted function of the encoded protein can help decipher the link between genotype and phenotype.

A range of *in silico* approaches can be used to assess the likely deleteriousness of a variant at the genetic level and are routinely incorporated into bioinformatics pipelines for WGS data analysis. These include CADD, PolyPhen-2, SIFT, MutationTaster and subRVIS (reviewed in 5). Various parameters are considered by these scoring tools, including sequence homology, evolutionary conservation, and elements of protein structure. Databases of genetic variants can also be highly informative: ClinVar^6^ annotates known missense variants for pathogenic or benign status whilst gnomAD^7^ aggregates data from a range of large-scale exome and genome sequencing initiatives, such as the 1,000 Genomes Project,^8^ highlighting variants that may be common in the healthy population to be considered causative for a rare disease. Furthermore, an absence of gnomAD variants in a region of interest may indicate that the gene may be intolerant to mutations.

The aforementioned tools assign a predicted severity score but do not suggest what the effect is at the protein level. Furthermore, some cases have been reported where the CADD scores do not correlate with disease severity.^9^ This discrepancy can often be rationalised by inspection of the protein structure. For example, an inverse correlation was found between CADD score of variants in the human RNA polymerase II subunit RPB1 (encoded by the *POLR2A* gene) and the severity of the associated neurodevelopmental phenotype. Variants expected to retain the ability to form stable subunit complexes were found to be more deleterious than truncations,^9^ most likely due to their sequestration of other components, such as RPABC3 (*POLR2H*), which is required by all three polymerases.

It is therefore important to assess the effect of an amino acid substitution at the structural level to understand its effect on protein function and the associated phenotype. A potential first step in assisting in the formulation of a hypothesis of the mechanism of any associated functional effect is to visualise the structural location of the target variant. Whilst 20% of the residues in the human proteome are covered by an experimentally determined structure, a further 30% are accessible via homologues.^10^ Recent machine learning advances (AlphaFold2^11^ and RoseTTAFold^12^) enable many more structured proteins to be reasonably modelled, providing additional opportunities to consider the structural impact of variants. Many tools are able to show the location of a submitted variant on a given structure whilst some online tools, such as MISCAST^13^ and Cosmic3D,^14^ identify on a given structure the location of residues altered by known variants in the human population. Cosmic3D provides an interactive interface that allows the user to click on a given variant in the feature tracks resulting in the display of a simplistic model of the variant. However, it is limited by its restriction to experimentally determined structures deposited in the PDB and known cancer variants (Cancer Gene Census), meaning not all variants of interest to the user can be displayed.

### Protein structure destabilisation

The destabilisation of protein tertiary and quaternary structures is the main contributor to variants' functional effects in around 50–70% of known pathogenic cases.^15^, ^16^, ^17^ Due to the complexities of its calculation,^18^ this has been a major focus of research in the literature.

Although the resulting change in protein function cannot be precisely predicted, it is possible to estimate the difference in relative Gibbs folding potential (ΔΔG) between the mutant and wild-type proteins using force-field–based molecular mechanics or statistically derived models. Several web and software applications exist that employ molecular mechanics to varying degrees. These are computationally expensive and provide only estimates of the effect due to complex technical limitations and assumptions, such as their use of a static structural snapshot and implicit solvent or force-fields that are imperfectly calibrated or too simplistic. Full force-field single-state calculations can be performed with the Rosetta suite^19^ or using FoldX.^20^ STRUM uses the I-Tasser algorithm for structure refinement to predict the best conformation of the variant and calculate its ΔΔG.^21^

A wide range of machine-learning–derived statistical models have been developed to address the issues around ΔΔG estimate calculation speed and accuracy. These include CUPSAT, SDM, DUET, mCSM, SNPMuSiC, MAESTROweb, pPerturb, MutaFrame and INPS-MD^22^, ^23^, ^24^, ^25^, ^26^, ^27^, ^28^, ^29^; reviewed in 30. Some, such as DynaMut, generate a consensus from different approaches.^31^ Recently, a second-order equation using just two structure-independent and one structure-dependent (relative solvent accessibility) variables was demonstrated to predict ΔΔG with competitive accuracy, suggesting that a small number of parameters provide significant information.^32^ However, these approaches do not output a 3D model of the mutations that can be visually inspected.

Missense3D avoids the need for ΔΔG calculations by flagging whether the variant matches any one or more of multiple criteria known to be destabilising,^33^ such as a proline residue located in an alpha helix, loss of key cysteines involved in disulphide bonding or a change in charge for a buried residue. Arguably this approach may be more intuitive than a simple numerical ΔΔG value. However, this tool only provides information relevant to structural stability and does not provide information on nearby variants from the human population or position-based annotations, which can play an important role in assessing functionality of variants. This is a limitation of existing ΔΔG calculation tools.

A recent article comparing these various methods with the aim of classifying pathogenic variants^17^ found a high false discovery rate and a low true positive rate. The best ranking method, FoldX, outperformed other methods but presented a false discovery rate of 35% and a true positive rate of 60% with a threshold of 1.58 kcal/mol. This may be explained by the observations that different structural domains have different cellular functions and tolerances to destabilisation and, critically, that pathogenic variants may exert their effects through a molecular mechanism other than stability.

### Beyond destabilisation

As discussed, it is known that the equivalence between destabilisation and altered protein functionality is only partial^15^, ^16^, ^17^ and the presence of proximal destabilising variants from the healthy human population (collated in the gnomAD database) may exclude the likelihood that such variants cause rare disease via a destabilisation mechanism. Other pieces of information including nearby bound-ligands or cofactors, post-translational modifications, presence of disulfide bonds, location within a transmemembrane span and sequence motifs (*e.g.* protein localisation signals) instead provide improved insights. Whilst a variant may result in decreased functionality (*e.g.* catalysis, signalling or sequestration) equivalent to a decreased protein concentration, it may also result in an increase in effective protein concentration by means of decreased degradation, altered localisation, diminished interactions or loss of regulation.

Different variants of the same protein can result in different pathogenic phenotypes. For example, dominantly inherited variants of LZTR1 result in a severe form of the developmental disorder, Noonan syndrome. These variants are predominantly located in the binding interface between LZTR1 and HRAS. In contrast, recessive destabilising variants result in a milder phenotype.^22^, ^23^

Structurally destabilising variants may have a dominant effect if the protein is affected by haploinsufficiency or by imbalanced inhibition as seen with G-protein β2,^35^ but often the variant has a recessive phenotype. However, *de novo* variants may result in gain-of-function, such as loss of regulation from a post-translational modification site (PTM). Over 1,950 known cases of pathogenic variants that affect a PTM are known^36^ illustrating the importance of considering non-structural effects in annotation of variants.

UniProt^37^ is an invaluable resource which aggregates various sources of curated information such as domain details, experimentally validated post-translational modification sites, signals, catalytic residues, transmembrane spans, and so forth, and can be used to investigate this additional layer of possible effects on protein function. However, many variants from WGS studies will be within proteins of unknown function which have been poorly characterised; in this situation uncurated and predicted information becomes highly valuable. For example, the PhosphoSitePlus database^38^ includes both PTMs identified from high throughput screens as well as well characterised sites. Similarly, the Eukaryotic Linear Motif (ELM) database may reveal if a residue span is within known motifs such as those determining protein localisation, or within a recognised cellular protein interaction site.^39^

A further limitation of available online tools to investigate the effect of a variant is the requirement for the researcher to possess significant structural biology expertise, including knowledge of how to obtain the most appropriate experimental model from the PDB^40^ or from online methods or repositories of predicted structures (*e.g.* Phyre2,^41^ I-Tasser,^41^ EBI–AlphaFold2^11^) for the protein in question. The analysis may be further challenged by the possibility of inconsistencies between the numbering of residues within the structural model and that in the context of the expression construct or whole-protein sequence. Although several tools exist, there are, presently, none that have the desired range of annotations for variants of interest which can be presented in an interactive manner to non-structural biologists.

### Venus – An interactive tool

**Figure 1:**
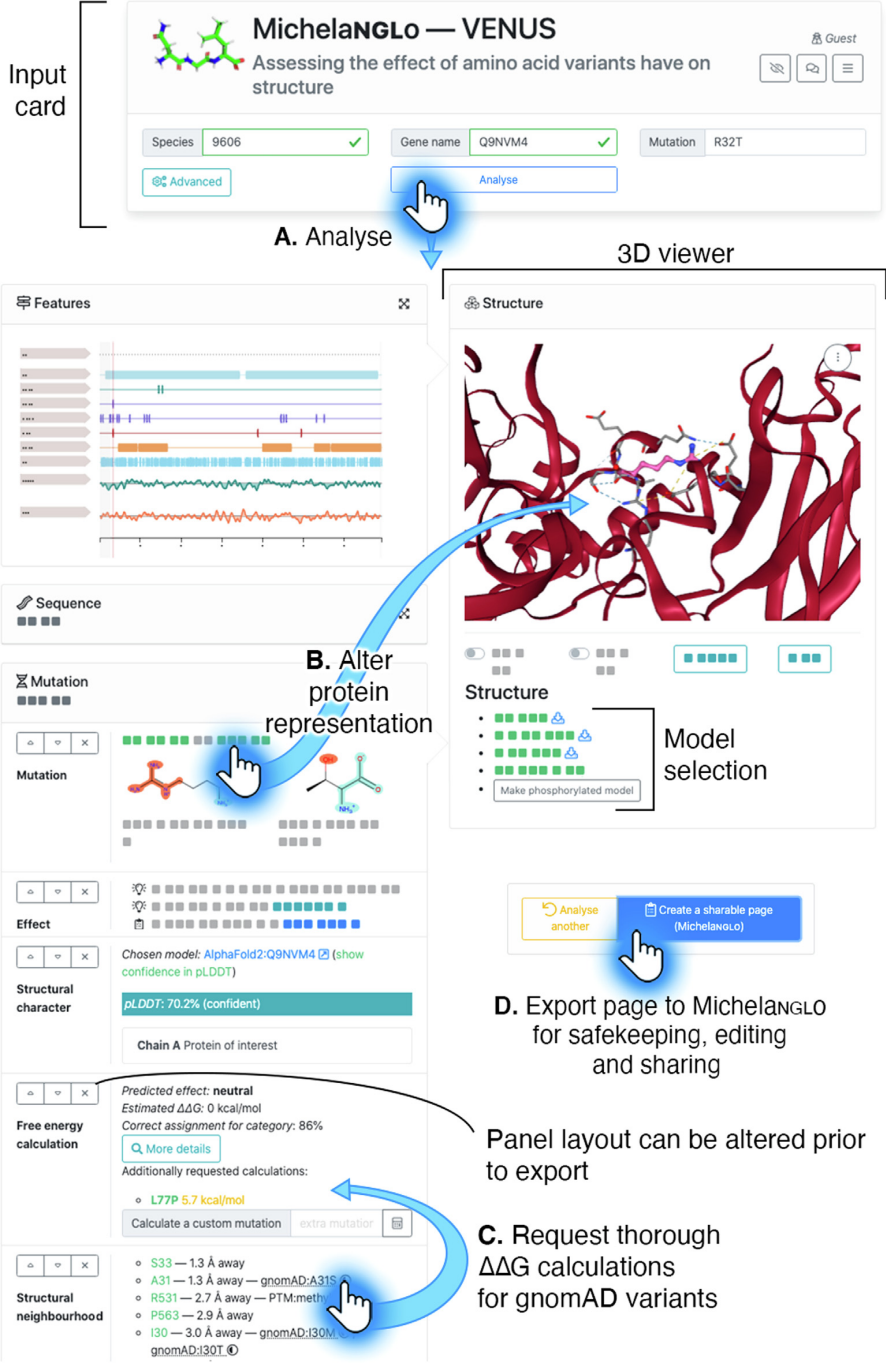
Layout and functionality of Venus. **(A)** The first step requires the user to provide the species, gene name or UniProt accession and the mutation of interest. Optionally, other settings may be altered, such as providing a custom model structure. **(B)** The cards on the left-hand side of Venus (simplified for illustrative purposes) contain links that control the 3D visualization within the NGL viewport present in the right-hand side card. **(C)** An estimate of the ΔΔG for additional variants from gnomAD can be calculated on request. **(D)** The page can be exported to a Michelaɴɢʟo page, which can be further edited and shared.

To address many of these challenges, we have developed **Venus** (https://venus.cmd.ox.ac.uk), a web application that, for a given species, protein name and protein substitution of interest, retrieves a suitable protein structural model and estimates the ΔΔG for that variant as well as any nearby known variants, and provides annotations for these neighbours which may impact the function of the protein. All of these annotations can be clicked upon within the interface resulting in their focus in the protein view (Figure 1).

## Results

### The Venus application

Venus is a web-based tool providing rapid access to information concerning a protein substitution in terms of the impact on predicted stability and protein features. Venus proceeds via several guided steps and displays the results to the user as these steps are completed, allowing initial inspection to immediately occur pending further analyses (Figure 1). Firstly, upon a valid input, non-structural data is shown from UniProt and ELM. Subsequently, the most suitable structural model is automatically chosen and shown. The residues within a 12 Å radius of the residue of interest are enumerated and annotated with information from different sources (*vide infra*). Meanwhile, the ΔΔG is estimated for the variant. Finally, the ΔΔG is also estimated for any nearby gnomAD and ClinVar variants. Additionally, on request, a more precise ΔΔG estimation for a specified variant can be calculated or a post-translationally modified model can be generated.

Because Venus utilises Michelanglo,^42^ interactive views and descriptions of the results of Venus can be created, shared and used collaboratively without requiring the user to have expertise in structural biology or protein informatics. Michelanglo has been shown to be of great utility by virtue of being able to clearly convey information in a more intuitive and interactive manner than an information-heavy and flat representation of a 3D structure. Several diverse uses of Michelanglo have been described, ranging from demonstrating the location of rare mutations to providing the active site configuration for biocatalysis and drug design applications.^35^, ^43^, ^44^, ^45^

### Protein structure model choice

**Figure 2:**
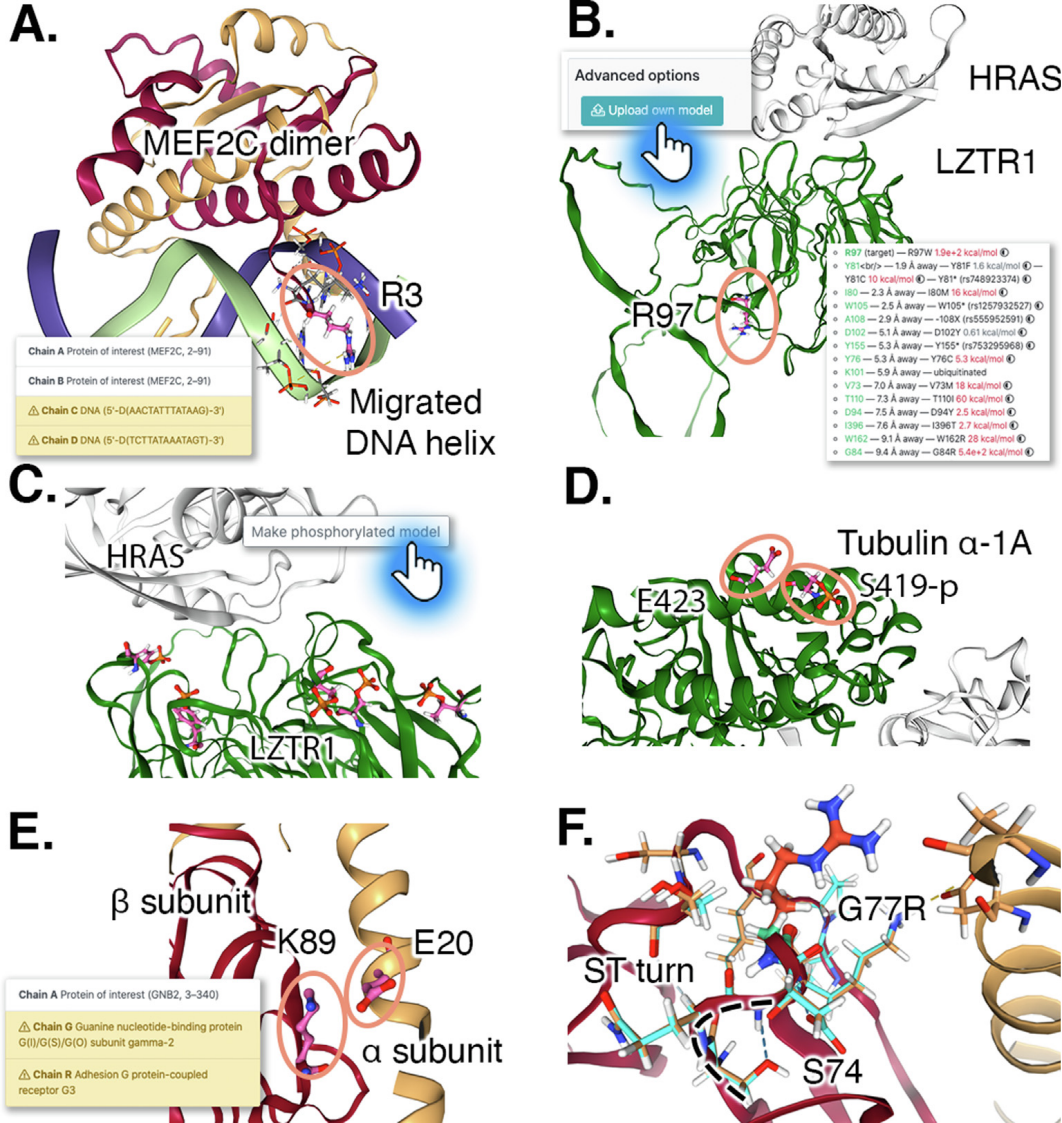
Examples of variant impact analysis with Venus, illustrating user-focused features and residues investigated circled. **(Panel A)** The effect of certain variants may be best interpreted in the context of a protein’s known binding partners: portraying MEF2C as a homodimer with the DNA copied from the MEF2A template reveals that the R3C substitution affects DNA binding and nucleotide specificity. In the structural information element of the left-hand side card of Venus the chains are listed and the copied chains flagged for further consideration (inset). **(Panel B)** The models available may not always be ideal and in certain cases providing Venus with a custom model is important to investigate a variant, as illustrated by the LZTR1:HRAS complex. Furthermore, the presence and effect on stability of nearby gnomAD variants may help formulate a hypothesis. In the case of LZTR1 R97L, these reveal that it is not an interface residue and that most gnomAD variants are highly destabilising, including R97W, in contrast to R97L, which is near neutral. **(Panel C–D)** Several variants are adjacent to phosphorylated residues, therefore it is important to have the option to make a model of these, as seen for the LZTR1 interface and Tubulin α-1A E423G, which is close to S419, a target of phosphorylation. **(Panel E)** In Venus, emphasis is placed on user inspection and interaction, as opposed to giving a single metric. The potential effect of certain variants may be multifaceted, for example in G-protein β2, subunit K89 forms a salt bridge with E20 of the α subunit (migrated chains in insert), but the substitution to threonine has a compensating stabilising effect, resulting in an overall neutral ΔΔG, furthermore, the residue is a ubiquitination target. (**Panel F)** The inspection of the overlay of models for wild type (teal/turquoise) and variant G77R (coral/gold) of the G-protein β2 subunit allows the formulation of the hypothesis that the G77R substitution in G-protein β2 subunit may affect the conformation adopted by the phosphorylation of S74, even if this is not available.

An important requirement is the identification of the most suitable structural model. These may be structures from the PDB^40^ (with any numbering offset corrected), Swiss-Model homology models,^46^ AlphaFold2 models^11^ or a user-provided models. A structure from the PDB is the preferred choice, if available. Warning flags may be displayed within Venus informing the user of the quality of the chosen model, such as poor-quality metrics for Swiss-Model (Q_mean_ < –2. or identity < 20%) and AlphaFold2 models (pLDDT < 70%). Where multiple protein chains are to be considered, Swiss-Model is used rather than AlphaFold2 because AlphaFold2 does not by default generate quaternary structures. This approach enables Venus to present the location of binding partners to the user. This was found to be a beneficial approach with MEF2C (Figure 2(A), 97% identity to the crystallised MEF2A, PDB:3KOV), where the pathogenic mutations previously reported^47^ fall broadly into two categories; those that are structurally deleterious (for example S36R) and those affecting DNA binding, several of which are not destabilising overall (for example R3G, Figure 2(A)): results which would not be apparent without the DNA being present in the visualisation.

For more complex use-cases, a model structure can be uploaded by the user (Figure 2(B)). LZTR1 provides an example of this, where an AlphaFold2 model is available, but a Swiss-Model structure at 19% sequence identity is excluded under default settings. To further investigate the binding hypothesis, an LZTR1:hRAS dimer model was predicted via ColabFold,^48^ a variant of AlphaFold2,^11^ and uploaded into Venus. Venus demonstrates that, except for R97L, the dominant variants of LZTR1 are clustered on one face of the β-propeller, which has been hypothesised to be the face where HRAS binds.^34^, ^49^ Venus’s estimation of the ΔΔG for the gnomAD variants near R97L indicate that they are likely to be highly destabilising (Figure 2(B) inset), consistent with the hypothesis that destabilisation is not the reason that the pathogenic *de novo* variants are deleterious.^34^, ^49^ Additionally, Venus reveals that several of these pathogenic variants in the interface, such as S244C, affect residues which are close to, or are themselves, residues found to be phosphorylated in high-throughput screens reported in the PhosphoSitePlus dataset.^38^ Furthermore, an interactive visualisation of the model of the residues predicted by PhosphoSitePlus to be phosphorylated is made available (Figure 2(C)). As a result of the Venus analyses, one may formulate a hypothesis that disrupted phosphorylation of LZTR1 may play a role in the pathogenicity, an interesting unexplored avenue of research.

### Free-energy estimations

For the structural analyses of the impact of protein substitutions on stability, two sets of benchmarks were undertaken. The first benchmark was to determine the accuracy of Venus’ ΔΔG estimations against two datasets, the second the failure rate.

Venus gives two ΔΔG estimations. The first is a near-instantaneous estimation using the second degree equation from 32. The second uses a molecular mechanics approach. The latter ΔΔG estimations are performed using PyRosetta via a protocol streamlined for speed. A force-field–based method was chosen because this also provides a model of the variant with not only the sidechain of the substituted residue altered, but also with nearby sidechains repacked and backbones moved. Venus energy-minimises residues within a pre-set radius of the target residue (for one or more cycles of FastRelax mover), introduces the mutation, and minimises again. This neighbourhood approach is more appropriate than naïvely picking the rotamer with the least pronounced degree of clash with neighbouring atoms.

To determine the optimal balance of speed and accuracy using different settings, predicted ΔΔG values were compared with empirically determined ΔΔG values. Public databases exist that have significant quantities of thermodynamic data, most notably ProTherm, ProThermDB and ThermoMutDB.^50^, ^51^, ^52^ However, the data is biased in composition (solvent exposure, secondary structure, amino acid composition *etc*.), therefore subsets are generally taken which yield different scores on benchmarks depending on the subset adopted. Three benchmark subsets were used that are filtered to be less biased and possess a structure from the PDB. These were ProTherm* (768 variants across 84 structures, ΔΔG: mean 1.0 kcal/mol),^54^ O2567 datasets (2567 variants across 106 structures, ΔΔG as ΔG_mutant_ − ΔG_wildtype_: mean 1.0 kcal/mol)^55^ and S1342 (1342 variants across 131 structures)^53^ (Results in SI Table 1).

Venus does not correct substantial backbone alterations that might be induced by protein substitutions relative to the wild type and as a result may overestimate the deleterious effect of certain variants. In these circumstances the values are shown to the user as “>10 kcal/mol”, an arbitrary cut-off close to the upper outlier cut-offs (Tukey upper fence) of the distribution of experimental values, which varies between 8 and 13 kcal/mol depending on the dataset and settings adopted.

Based on the benchmarking tests, the chosen default settings were two minimisation cycles under the standard Rosetta scorefunction (ref2015),^19^ targeting all neighbouring residues whose Cβ atoms are within a 12 Å radius of the target residue. This calculation takes under 30 seconds for all three datasets. Under these settings between 62% (S1342, φ coefficient: 0.36) and 71% (ProTherm*, φ coefficient: 0.43) of samples were predicted to result in a ΔΔG greater or lesser than 2 kcal/mol concordantly with the experimental values. For the S1342 dataset under the default conditions the median absolute error is 1.2 kcal/mol, whilst the Pearson correlation coefficient, after the exclusion of outliers given the aforementioned inaccuracy at higher values, was 0.21 and the mean absolute error 1.7 kcal/mol. The correlation increases to 0.43 when the settings are altered (5 cycles under the cartesian beta2016 scorefunction), but this results in an increased calculation time (median from 24 seconds to 170 seconds) and does not offer an increase in accuracy in classification around the 2 kcal/mol threshold. Nevertheless, the settings used by Venus can be altered by the user both in terms of model choice and ΔΔG calculations.

Venus aims to be able to analyse any given proteins, hence its use of Swiss-Model and AlphaFold2 models. The ΔΔG for variants in the O2567 dataset was scored using either a Swiss-Model or an AlphaFold2 model instead of the available PDB structure. This resulted in similar errors, but slower calculation times (median times: 19, 21 and 27 seconds for PDB, AlphaFold2 and Swiss-Model, SI Table 1) which may be considered to be acceptable in terms of user experience. The ProTherm*, O2567 and S1342 datasets contain high-quality single chain crystal structures, whilst the structure or model chosen within Venus may not meet these quality criteria (*e.g.* very large assembly, low resolution, distorted sidechains). To explore whether these may fail or cause an increase in calculation time, 300 randomly generated protein substitutions in different human proteins were tested (SI Table 2). The ΔΔG calculations were completed for all substitutions, with 85% being completed in under one minute whilst for five proteins, all components of large complexes, the calculations took over 5 minutes.

### Neighbourhood features

An important feature of Venus is its ability to provide the user with information concerning the neighbourhood surrounding the target variant. Detailed annotations are provided for residues within 10 Å of the variant of interest. This includes (i) conservation information (in the case of structures from the PDB and Swiss-Model-sourced structures, this is expressed as normalised score from ConsurfDB), (ii) entries in gnomAD or ClinVar databases, (iii) post-translational modifications and (iv) overlapping features reported in UniProt. These residues, along with other regions mentioned in the results, can be clearly displayed in 3D by clicking on their green links.

An example of the utility of this approach is furnished by α-tubulin 1A (TUBA1A) E423G (Figure 2(D)), a novel *de novo* variant identified in the OxClinWGS WGS dataset.^2^ This variant is neutral in terms of stability but is 2.0 Å away from S419, a phosphorylation site and is in a neighbourhood devoid of variants reported in gnomAD. Another example is G-protein subunit beta-2 (GNB2 encoded) K89T^35^ (Figure 2(E)), a mutation predicted to be mostly neutral in terms of stability, but is a ubiquitination site and interacts with the alpha subunit. The ability to visually inspect the variants is helpful because in some cases the interpretation is not straightforward. For example another G-protein subunit beta-2 variant, G77R^35^ (Figure 2(F)), also neutral in terms of structural stability, is proximal to two phosphorylated residues (S74 and S76) but not facing them. On visual inspection, G77 can be seen to be part of an Asx turn, which might be affected by the G77R variant. This is followed by an ST turn involving S74, which suggests its phosphorylation may alter the local structure, resulting in a change in protein function, suggesting why the variant was found to be pathogenic.

To quantify, from a global viewpoint, the frequency of pathogenic or benign variants in large datasets, the ClinVar dataset and the nearby gnomAD variants were scored with Venus (SI Table 2). The analysed subset of ClinVar variants with a pathogenic consequence (9,960) contained 3.5 times more variants with a ΔΔG greater than 2 kcal/mol than the subset with a benign consequence (14,414), but this accounted for only 19% of the subset. However, only 3 of these destabilising pathogenic variants (1,909) were within 10 Å of a predicted destabilising gnomAD variant that was found in the population in a homozygous state or with a frequency greater than 5x10^-4^. This contrasts with the benign variants predicted to be destabilising for which over half (519 out of 797) were with 10 Å of a predicted deleterious gnomAD variant with high frequency. It is important to note that the ClinVar dataset is biased towards recurrent variants, and *de novo* variants may be under-represented, therefore the distributions are indicative only. Nevertheless, this demonstrates the utility of nearby variants to either lend support or disprove a destabilisation hypothesis for the cause of pathogenicity of a variant.

Enrichment of other features provide possible explanation for the cause of pathogenicity. Relative to benign variants, pathogenic variants were 4.5-fold more abundant within 10 Å of a ligand or cofactor (11% of pathogenic variants) or an interface (16% of pathogenic variants). The most abundant features observed were post-translational modifications, which were within 10 Å for 54% of the pathogenic variants and 37% of the benign variants. This difference is modest and reflects the fact that most post-translational modification may have little to no role in protein function, whilst a small minority may be critical for conformational switching or enabling the binding of other proteins. By presenting possible contributors to destabilisation, Venus provides opportunities to explore these and support further hypothesis generation.

## Discussion

Our investigations of potential pathogenic variants from large genome sequencing projects aimed at providing genetic diagnoses for patients with rare diseases, such as WGS500,^1^ OxClinWGS,^2^ DDD study^3^ and Genomics England’s 100,000 Genomes Project (100 kGP),^4^ have frequently required detailed annotation of these variants to inform assessment of their functional effects, beyond a predicted genetic pathogenicity score. Venus was developed in close collaboration with geneticists and several decisions in its developments were steered by this interaction.

Venus provides an interactive visualisation of a structural model of the variant for inspection, in context with other interacting proteins where known, along with location of residues that have non-structural functional roles (Figure 1). It provides the user with multiple pieces of information about the neighbourhood which the user can explore interactively and interpret. The user is guided into further investigating the information assembled by Venus by visiting the source of that piece of information. Venus therefore supports hypothesis generation rather than confirming a hypothesis of pathogenicity, which must be separately confirmed by functional studies.

A forcefield method was adopted for the estimation of the ΔΔG of a given protein substitution in order to be able to display a plausible structural model. Nearby sidechains and backbones may be shifted with this approach as opposed to a simple selection of a rotamer of the target residue, which may result in artifactual clashes. On average, the ΔΔG estimation is complete within 30 seconds. But since Venus presents results sequentially, rather than all at once, the wild type structure visualisation is quickly displayed in an interactive form for inspection pending the ΔΔG estimation being completed.

Whilst the error of the ΔΔG estimations for the highly destabilising variants is relatively high, the overall error is comparable to other methods when removing outliers or using median based metrics. The median absolute error is 1.1 kcal/mol for the S1342 dataset. In context, 1 kcal/mol is approximately the strength of a hydrogen bond and the cut-off for a destabilising variant is generally taken to be 2 kcal/mol. Many machine-learning–derived models possess intrinsic cut-offs for the maximum calculated ΔΔG value. For example, the SIMBA-I second degree equation^32^ cannot exceed +1 kcal/mol for a surface residue and +4.5 kcal/mol for a buried residue, whereas in a molecular mechanics system the forcefield has no such limits and the energy minimisation sampler/mover may be unable to escape a local minimum. A significant advantage of these two approaches is their delivery of a model structure for investigation, which may have nearby residues repositioned to accommodate the change.

The goal of Venus is to provide the user with multiple pieces of information about the neighbourhood which can be explored interactively and interpreted. The estimated ΔΔG of the protein substitution is not the sole possible determinant of pathogenicity. Our global survey of pathogenic and benign ClinVar variants found only 19% of pathogenic variants to have a ΔΔG greater than 2 kcal/mol (35% at >1 kcal/mol and 67% at >0 kcal/mol). When the estimated ΔΔG values of nearby variants from gnomAD were considered, the difference between pathogenic and benign ClinVar variants becomes more apparent. Additionally, the details of the system become important when considering variants case-by-case, as demonstrated in the examples presented.

Our investigations of the rare variants emerging from the OxClinWGS WGS dataset^2^ have shown that, even though changes in protein structural stability were the most common cause of pathogenic recessive variants, certain mutations which were deemed structurally neutral were found to affect a protein interface or other feature of interest. Therefore, other functional effects may be contributing to these non-destabilising cases. Venus gives an indication of what these may be. An example of this is the aforementioned example, α-tubulin 1A (TUBA1A) E423G (Figure 2(A)), which is close to a potential phosphorylation site, which may be involved in protein–protein interactions; a literature search reveals that S419L is pathogenic,^56^ further giving support to the hypothesis that destabilisation may not be the cause of pathogenicity.

Venus supports the exploration of proteins where information may be limited, as is often the case with WGS datasets which lend themselves to novel gene discoveries where the encoded proteins have been poorly characterised. Protein partners may be included from the template structure in Swiss-Model threaded models and post-translational modification detected solely in high-throughput screens can be used. The examples of MEF2C R3G and S36F^47^ (Figure 2(A)) and G-protein beta-2 K89T and G77R^35^ (Figure 2(B + C)) demonstrate that the model presented can be properly contextualised, even if no crystal structure is available. Nevertheless, the model may represent only one of several conformations, may be imperfect or may lack important binding partners, so consequently custom models can be uploaded as demonstrated with LZTR1 R97L^34^ (Figure 2(B)).

Substitutions of surface residues involved in protein–protein interactions are a very important class of pathogenic variant. However, Venus is currently unable to provide information on protein-binding sites without empirical evidence for the site of interaction. For some protein–protein interactions there are experimental complex structures available, but in most cases the precise structural detail of an interaction is not known. Enhanced evolutionary conservation of the residues may provide some indication of an interaction. MutPred2, a deep learning algorithm, is able to assign the probability of a residue being involved in an inter-molecular interaction from the primary sequence context.^57^ However, without knowing the binding partner, the researcher is limited in the functional studies that can be undertaken.

Whilst for post-translational modifications high throughput data is used in Venus to complement the curated data in Uniprot, there is presently no mature dataset for protein–protein interaction sites. The most applicable high-throughput technique to identify the precise location of a protein–protein interaction are untargeted cross-linking mass-spectrometry (XL-MS) techniques,^58^ which, due to the associated technical challenges, have so far been of limited use and a low sensitivity. As a result, Venus does not utilise this information. Nevertheless, the data provided, such as the conservation and nearby gnomAD variants, may help the user determine what may be the role of the region.

One future feature that would be useful for Venus is the consideration of alternative conformations. AlphaFold2 has prompted a flurry of research in a variety of directions, including modelling of alternative states of proteins and protein complexes, including conformers that may be transient.^11^, ^48^ Currently, there are a limited number of PDB structures in alternative states and EBI-AlphaFold2 provides only one single-chain model per protein. However, it can easily be envisaged that a database of human oligomeric proteins in alternative conformations may arise in the future. This would be a great boon to Venus as currently the user has to identify or create a structure or model of an alternative state and upload it to Venus, as was shown for LZTR1.

## Conclusion

Venus integrates multiple sources of information to aid in the interpretation of the effect of a genetic variant on the function of its encoded protein. By presenting information concerning protein structure, energies of destabilisation, effects on post-translational modifications and protein interaction sites, and displaying these in the interactive Michelanglo application, Venus extends the analyses possible with existing tools. We anticipate that this will be a valuable resource for helping geneticists and other scientists investigate the potential effects a variant of interest is having on protein function and hence its likely pathogenicity when studied in the context of patients with rare diseases.

## Materials and Methods

Venus is built into Michelanglo and the codebase is openly available in GitHub (https://github.com/CMD-Oxford/Michelanglo-and-Venus). Michelanglo is a Python 3 webapp running the Pyramid framework with a PostgreSQL database for user data.

Venus aggregates information from UniProt entries with data derived from various sources. UniProt is parsed for sequence and feature information,^37^ gnomAD for healthy human population variants,^7^ PhosphoSitePlus for post-translational modifications found in high throughput studies,^38^ SIFTS data for PDB numbering correction,^59^ and the RCSB for PDB metadata.^40^ For the predictions of loss or gain of linear motifs spanning the mutation, the regular expression patterns from ELM^39^ are searched.

During structure model selection, Venus takes experimental crystal structures with the best resolution deposited in the RCSB PDB,^40^ if they exist. If no solved structures are available Venus uses a model from Swiss-Model^46^ within a user-specified sequence identity cut-off. Otherwise an AlphaFold2 model is retrieved.^11^ If this is not possible, only structure-independent information is provided to the user. Once a candidate model is chosen, it is obtained from the relevant location and modified with PyMOL. PyMOL is used to correct the residue numbering offset for the model structure, to rename the chain in question to ‘A’ and to remove solvent and common crystallisation-derived small molecules using a modified list taken from 60. For Swiss-Model structures, any other chains present in the template are copied unless steric clashes are present. For PDB and Swiss-Model structures, ConsurfDB is queried for the conservation data and then applied as B-factors to these.^61^ The ΔΔG estimations are performed in PyRosetta using the FastRelax mover^62^ targeting only the local neighbourhood.

In the web interface, the protein structure is visualised using the NGL JavaScript library^63^ and the features and sequence are shown using the NeXtProt viewer JavaScript library.^64^ Documentation and video tutorials are available via the Venus web interface.

In addition to browser-based access, Venus can also be queried computationally with a client-side Python API (pypi: michelanglo-api). To assess the frequency that Venus successfully completes a requested analysis, 300 random protein substitutions were requested via the API (summary results in SI Table 2).

To determine the optimal settings for energy minimisation for ΔΔG calculations, mutations from the ProTherm*,^54^ O2567^53^ and S1342^55^ datasets were scored using a range of different parameters (summary results in SI Table 1, scripts, data and plots available at https://github.com/CMD-Oxford/validation_of_venus_ddG). Specifically, the protein analysis module of Venus was used in isolation on a computing cluster with different Rosetta forcefields (talaris2014, ref2015, beta_nov16), within cartesian or dihedral space, different number of FastRelax descent cycles (1–5), different neighbourhood radii (6–12 Å) and with or without minor correction artifices. These corrections were tested because the model structures are only energy minimised within a sphere of neighbours around the mutated residue. The primary focus of these was on the interactions between the outer neighbourhood shell to the residues beyond the shell, which were not energy minimised, but may have been energetically strained. These corrections included scoring only the minimised neighbourhood, constraining the residues at the neighbourhood interface, and preventing the acceptance of a poorer overall score caused by an improvement of a locally bad conformation. The median absolute error was calculated by taking the median of the absolute difference between the predicted and experimental ΔΔG values. The Tukey fences were calculated with a scaling factor of 1.5 (standard value). These were used to eliminate the outliers prior to the calculations of metrics thrown off by few spuriously large values, such as mean absolute error, root mean square deviation and Pearson correlation coefficient. The confusion matrices were cross-tabulated by rounding to one decimal digit the predicted ΔΔG values (to match the precision of experimental ΔΔG values) and by classifying the values for greater or equal to 0 kcal/mol or 2 kcal/mol.

ClinVar and gnomAD variants were scored using the protein analysis module of Venus (summary results in SI Table 3). All human protein were filtered for the presence of a ClinVar variant and further filtered against protein with submitted variants whose mutations were inconsistent with the canonical sequence (222). The ClinVar and gnomAD variants in the resulting protein list (354,546 in 9,123 protein) were scored and the output parsed to extract key details that would normally be shown by the front-end.

Venus is free to use without requiring user registration. Due to the licences associated with the datasets and modules used, the protein data is not disseminated in the repositories and commercial users must obtain licences from PyRosetta, ELM and PhosphoSitePlus prior to usage. Venus is intended for research and not diagnostic purposes.

### CRediT authorship contribution statement

**Matteo P. Ferla:** Conceptualization, Methodology, Software, Investigation, Writing – original draft, Visualization. **Alistair T. Pagnamenta:** Conceptualization. **Leonidas Koukouflis:** Resources. **Jenny C. Taylor:** Funding acquisition, Supervision, Writing – review & editing. **Brian D. Marsden:** Supervision, Writing – review & editing.

## Data Availability

Web app code and analysis data are publicly available in GitHub
